# Objective Characterization of Opiate-Induced Chest Wall Rigidity

**DOI:** 10.7759/cureus.8459

**Published:** 2020-06-05

**Authors:** Charles Trujillo, David Rudd, Hakan Ogutcu, Fanglong Dong, David Wong, Michael Neeki

**Affiliations:** 1 Surgery, Arrowhead Regional Medical Center, Colton, USA; 2 Emergency Medicine, Arrowhead Regional Medical Center, Colton, USA

**Keywords:** opiate, chest wall rigidity

## Abstract

Introduction

Opiate-induced chest wall rigidity is a syndrome that largely goes unrecognized. To date, no study has presented significant objective data to better understand this syndrome.

Objective

The aim of this study was to explore the correlation between the dosage of opiates and the incidence of chest wall rigidity, ventilatory changes, and effects of naloxone administration.

Methods

A total of eight patients were identified as having episodes of chest wall rigidity, with half of the population being females, with an average age of 54.8 ± 9 years. Physiological changes, ventilator data, vitals, and opiate dosage prior to chest wall rigidity episodes and after reversal with naloxone administration were analyzed using the Wilcoxon rank sum test for statistical significance.

Results

Significant changes were observed in dynamic wall compliance without positive end-expiratory pressure (PEEP) (pre-median=5.13; post-median=52.03; p=0.0078), dynamic wall compliance with PEEP (pre-median=6.13; post-median=72.36; p=0.0078), tidal volume (pre-median=110.5; post-median=1006; p=0.0078), and ventilator airflow (pre-median=0; post-median=75; p=0.0078). However, no statistically significant changes were detected in end tidal CO_2_ (pre-median=36; post-median=37.5; p=0.4219), respiratory rate (pre-median=9; post-median=10.5; p=0.7188), or peak airway pressure (pre-median=17; post-median=21.5; p=0.4063). Additionally, there is a statistically significant correlation between morphine equivalent potency dosing within 24 hours and the change in tidal volume (r=0.8237; p=0.0439).

Conclusions

Our study is the first to demonstrate significant objective data on the ventilatory responses seen with opiate-induced chest wall rigidity. These findings may assist clinicians in better understanding the presentation and management of chest wall rigidity.

## Introduction

Chest wall rigidity (CWR) is a phenomenon first described by Hamilton and Cullen in 1953 [1]. Characterized by extensive rigidity primarily in the musculature of the chest and abdomen, CWR can affect spontaneous ventilation due to diminished chest wall compliance [2]. Many proposed theories have attempted to explain the exact pathological mechanism behind CWR. No definitive mechanism has been proven; however, some experts argued that the process seems to be a central neurologically mediated process [2]. Animal studies have confirmed these theories, with implications on central nervous dopaminergic pathways as well as centrally mediated Mu opiate receptors [3,4].

Despite many hypothesized theories, the actual incidence of CWR is largely unknown. This is largely due to underreporting and the inability to properly identify patients who have experienced the syndrome. Since it was first described, numerous case studies have been published detailing the occurrence of CWR. Comstock et al. described extreme difficulty in ventilating cardiac surgery patients after the use of high-dose fentanyl during anesthetic induction [5]. Çoruh et al. described similar findings with versed and fentanyl during a routine bronchoscopy [2]. An additional case report detailed the same presentation in a healthy 22-year-old female with no comorbidities during a routine dilation and curettage, further highlighting the unpredictability seen with this syndrome [6]. However, the presentation of CWR is not always clearcut. The timing of opiate administration and actual manifestation of CWR can have a variable and delayed onset, which can further complicate the diagnosis [7].

To date, there are minimal literature and objective data available to characterize CWR. This case series aims to clarify the pathophysiological changes and response to treatment when faced with this syndrome. It is hoped that these examples will provide physicians with an objective method to identify and treat CWR in an effective fashion, with objectively derived factors.

## Materials and methods

This case review is a retrospective study that reviewed patients from 2014 to 2017 at Arrowhead Regional Medical Center, a level II trauma center located in Southern California. Electronic medical charts of patients who were on ventilator management were reviewed. Patients identified to have complicated or difficult ventilation were reviewed for physical presentation and ventilator findings indicative of CWR. Patient characteristics including age, sex, nature of admission, nature of injury, history of narcotic use prior to admission on urine drug screen, pattern of ventilator breathing, BMI, dose, and type of narcotic prior to the episode of CWR were assessed. Narcotic dosages were converted into morphine equivalent dosages to maintain consistency. Vital signs six hours prior to identified CWR episodes and two hours after naloxone administration were reviewed. Objective ventilator data including respiratory rate, peak airway pressure, dynamic compliance with and without positive end-expiratory pressure (PEEP), ventilator flow rate, tidal volume, and end-tidal CO_2_ during episodes of CWR and after administration of naloxone were recorded.

To obtain these data, ventilator waveform tracings during episodes before and after naloxone were plotted for statistical analysis. Descriptive statistics were presented as medians and interquartiles (first and third quartiles) for continuous variables, along with frequencies and proportions for categorical variables. The Wilcoxon rank sum test was used to analyze ventilator responses seen during CWR and after naloxone administration. Pearson’s correlation coefficient test was performed to assess for possible correlations between narcotic dosage given within 24 hours of CWR and ventilator variables.

## Results

A total of eight patients were identified to have episodes of opiate-induced CWR. Males and females were equally distributed, with an average age of 54.5 years (first quartile = 48 years; third quartile = 28 years). Patient characteristics are presented in Table 1. Statistically significant changes were found in dynamic wall compliance without PEEP (pre-median=5.13; post-median=52.03; p=0.0078), dynamic wall compliance with PEEP (pre-median=6.13; post-median=72.36; p=0.0078), tidal volume (pre-median=110.5; post-median=1,006; p=0.0078), and ventilator airflow (pre-median=0; post-median=75; p=0.0078). Additionally, there is a statistically significant correlation between morphine equivalent potency dosing within 24 hours of CWR episodes and subsequent changes in tidal volume (r=0.8237; p=0.0439). No statistically significant changes were detected in end tidal CO2 (pre-median=36; post-median=37.5; p=0.4219), respiratory rate (pre-median=9; post-median=10.5; p=0.7188), or peak airway pressure (pre-median=17; post-median=21.5; p=0.4063).

**Table 1:** Comparison of clinical outcomes between pre- and post-median. All values are presented as median with first and third quartiles in the parenthesis; p-values were calculated using the Wilcoxon rank sum test. PEEP, positive end-expiratory pressure

	Pre-median	Post-median	p-Value
Dynamic wall compliance without PEEP	5.13 (0.29, 10.86)	52.03 (37.99, 63.88)	0.0078
Dynamic wall compliance with PEEP	6.13 (0.43, 20.02)	72.36 (61.1, 92.73)	0.0078
End tidal CO_2_	36 (32.5, 48.5)	37.5 (33, 40)	0.4219
Respiratory rate	9 (8, 10)	10.5 (8.5, 13)	0.7188
Tidal volume	110.5 (5, 167.5)	1,006 (832, 1,174.5)	0.0078
Ventilator airflow	0 (0, 24)	75 (62.5, 90)	0.0078
Peak airway pressure	17 (16, 22)	21.5 (18, 25)	0.4063

## Discussion

Opiate-induced CWR is a syndrome that has limited reported understanding despite its appearance in multiple case reports. However, the pathophysiological mechanism behind this syndrome has yet to be defined. With a large amount of ambiguity, the medical community has yet to come to a consensus. Our study is the first to demonstrate significant objective data on the ventilatory changes seen with this syndrome.

Our case series expands on the consensus reported in previous literature. To begin, the airway and ventilatory response seen in airway compliance with the paucity of flow reached statistical significance in our study. Scamman characterized the main pathological finding for CWR episodes to be a reduction in chest wall compliance, as characterized in our results [8]. Scamman’s study demonstrated a mean reduction of 16% in static airway compliance, after fentanyl was administered during the induction of anesthesia [8]. However, the study sample only included five cases [8]. An animal model analyzing fentanyl responses in rabbits also reported a similar response with a mean decrease of 22% in airway compliance, which was seen in 5 of 11 subjects [4]. Additionally, a small cohort study by Klausner et al. reported the need for increased airway pressures to deliver adequate tidal volumes in patients experiencing this syndrome during induction [7]. Despite similar findings seen in chest wall compliance during our study, our study is unique in that it characterizes this syndrome in a context outside of induction. Our narcotic dose equivalency results also suggest that the effect is seen in the potency and not unique to fentanyl. Historically, CWR episodes have been reported to have profound hemodynamic effects. Though statistically significant changes have been reported in mean arterial pressure, central venous pressure, and pulmonary capillary wedge pressure, the cardiovascular response remains highly variable, with hypertensive, hypotensive, to negligible responses reported by numerous studies [2,9,10]. The hemodynamic changes in our study failed to meet statistical significance. The lack of these findings in our data may be due to charting error or documentation delay, as our facility mostly uses a paper-based medical charting system. Additionally, certain patients included in the study may have been on vasopresser support due to hypotension unrelated to CWR. Another consideration is that the hemodynamic effects of our patients were limited by our reversal of paralysis or by narcotics and pressure-limiting ventilator modes before hemodynamic effects can be seen, as in worsening intrathoracic pressure or narcotic effects. The hemodynamic changes in response to CWR may have further influencing factors including mixed ventilator settings, genetic, patient sensitivity to narcotics, and medical history. However, the findings in our case series are unable to either confirm or negate these possibilities.

The relationships between CWR, narcotic type, dosing, and duration have all been implicated in its severity. The majority of literature surrounding CWR lists fentanyl as the most common perpetrator [8, 9,11-13]. However, in contrast to the literature, in the majority of our study sample, we used morphine during observed episodes, another unique aspect of our findings. Scamman reported a mean fentanyl dose of 17 ± 3 mcg/kg (±standard error of mean) when difficulty in bag-mask ventilation was observed with a subsequent reduction in pulmonary compliance [8]. Klausner et al. reported a fentanyl “rigidity threshold” of 7 ml/L as a level at which CWR occurs. Comparably, our results also demonstrate a significant correlation in narcotic equivalent dosage 24-hours prior to CWR episodes and subsequent changes in tidal volume (r=0.8237; p=0.0439). The delay in presentation seen in our experience has been described as well with delayed effects reported up to six hours after administration depending on dosage [7]. The characteristic muscular rigidity seen with difficulty in ventilation and markedly increased airway pressure during these episodes has been extensively described [5,7,12,13]. This rigidity was observed during clinical evaluation of all patients included in our study. The rigidity and subsequent ventilatory changes have been widely hypothesized to be a central process related to Mu dopaminergic neurons [3,4]. Other studies have proposed glottic closure and upper airway obstruction as the proposed mechanism for its pathophysiology [8]. However, this clinical presentation was still seen in intubated patients. We instead found chest wall and abdominal rigidity resulting from a very prolonged forced exhalation phase. The increase in airway pressure reported in the aforementioned studies was controlled for in our patients, as all patients were on pressure-controlled ventilation. We instead observed reduced, and at times, paucity of inspiration with reversal of air inflow during CWR episodes. This, as well as the findings with the narcotics other than fentanyl as a causative agent, suggests that this phenomenon may be a spectrum of narcotic overdose affecting centrally and not peripherally the muscle receptor interface.

Without the historically reported changes in airway pressure seen in previous studies, the actual presentation of CWR, in part, may be a misnomer. Our theory is that CWR may, in fact, result from an active process of prolonged forceful exhalation that limits airflow with a subsequent relaxation in the musculature and resumption of airflow. This characteristic pattern perpetuates until the inciting medication effect is reversed. A French study proposed similar thoughts implicating GABAergic inter-neuron disinhibition and eventual ventral horn activation as the proposed mechanism behind CWR [14]. Neidhart et al. also corroborated the analogous view that though the inciting effect leading to CWR may be centrally-mediated, the ventilatory effects responsible for its pathology, in fact, may be due to an active response induced by the thoracoabdominal musculature, as symptoms abate upon relaxation [9].

## Conclusions

The incidence and mechanism behind CWR secondary to opiates are largely unknown. The results of our study are paramount, as the ventilatory pattern seen in our study controlled for airway pressure. The observations seen in our case series show a reduced pattern of airflow without subsequent pressure increases. This suggests that CWR due to opiate administration may be caused by an apneic-type response with prolonged forceful exhalation that is active and not entirely centrally-mediated. Given our small sample size, further studies are needed to confirm these findings.
